# The Influence of Vaginal HPV Self-Sampling on the Efficacy of Populational Screening for Cervical Cancer—An Umbrella Review

**DOI:** 10.3390/cancers14235913

**Published:** 2022-11-30

**Authors:** Tomasz Tatara, Katarzyna Wnuk, Wojciech Miazga, Jakub Świtalski, Dagmara Karauda, Paulina Mularczyk-Tomczewska, Urszula Religioni, Mariusz Gujski

**Affiliations:** 1 Department of Public Health, Faculty of Health Sciences, Medical University of Warsaw, 02091 Warsaw, Poland; 2 Department of Health Policy Programs, Department of Health Technology Assessment, Agency for Health Technology Assessment and Tariff System, 00032 Warsaw, Poland; 3 Department of Health Economics and Medical Law, Faculty of Health Sciences, Medical University of Warsaw, 01445 Warsaw, Poland; 4 School of Public Health, Centre of Postgraduate Medical Education of Warsaw, Kleczewska 61/63, 01826 Warsaw, Poland

**Keywords:** cervical cancer, female cancer, screening, HPV, self-sampling

## Abstract

**Simple Summary:**

Cervical cancer (CC) is a common malignancy in the female population, resulting in a significant reduction in the quality of life and premature deaths. Since the prognosis depends on the stage of the disease, screening programs are of great importance in the management of cervical cancer. Several screening tests are available for this type of malignancy. Self-collection of vaginal samples for HPV tests is becoming increasingly popular; therefore, the objective of this umbrella review was to analyze the available data on their reliability. Secondary evidence from examinations of the sensitivity and specificity of the test, screening reportability data, the acceptance rates, and the cost-effectiveness of the method were taken into account. The review confirms the high usefulness of self-sampling in the early detection of CC as being due to increased uptake rates. Self-sampling is an effective and feasible modality for screening examinations which may be particularly important in the context of possible systemic changes in countries where CC screening is carried out by other methods and the screening uptake rates are low.

**Abstract:**

Introduction: Early detection of cervical cancer is a matter of great importance as the prognosis depends on the stage of the disease. The objective of the study consisted in the assessment of the impact of HPV self-sampling on the efficacy of populational screening programs aimed at early CC detection. Methods: The analysis was performed taking into account the Cochrane Collaboration guidelines for systematic reviews. The analyzed articles were searched for in the following databases: Medline (PubMed), Embase (Ovid), and Cochrane Library. Results: From a total of 60 citations, 16 studies were included in this review. The HPV test is highly sensitive and specific although the diagnostic accuracy of tests carried out in self-collected samples is slightly lower than that of tests carried out in samples collected by clinicians. The results of meta-analyses for HPV tests performed on self-collected samples indicate that the sensitivity for detecting CIN2+ ranges from 74% to 86% (depending on the publication and the analyzed population), and for CIN3+ from 75% to 86%. One publication showed a clearly lower sensitivity of 42% in detecting CIN3+, but the result is for a high-risk population and comes from only 1 RCT. The specificity of the assay exceeds 80% and 79.5% with regard to the detection of CIN2+ and CIN3+, respectively. As shown by the studies included in the review, both the offering of HPV self-sampling kits to patients and the mailing of such kits significantly increase the uptake of and participation in cervical cancer screening programs. In addition, self-sampling was found to be acceptable by the female subjects. Conclusions: HPV self-sampling is an innovative and cost-effective way to perform screening tests. In addition, self-sampling significantly increases the willingness to participate in screening programs among female subjects.

## 1. Introduction 

According to the estimates from the World Health Organization (WHO) and the International Agency for Research on Cancer (IARC), cervical cancer (CC) was the fourth most commonly diagnosed malignancy and the fourth most common cause of cancer-related death in women in 2020. A total of 604,127 (3.3) new cases and 341,831 (3.4) deaths were recorded in relation to CC that year although the incidence and mortality rates vary significantly according to geographical location (age-standardized in parentheses, incidence rate per 100,000 people, years based on the 1966 Segi-Doll World standard population) [1].

The Institute for Health Metrics and Evaluation presented the data on disability-adjusted life years (DALY) in relation to cervical cancer per 100,000 subjects in different age groups. DALY is a measure used to determine the patient’s health condition and represents the number of total years lost due to untimely death or disability due to injury or disease. In 2019, the highest DALY in relation to the disease of interest amounted to about 615.98 DALYs/100,000 women aged 55–59 years. A sharp increase in the DALY indicator is observed starting from the age of 30 (approx. 162.26 DALYs/100,000). This indicator remains relatively high in all older age groups, although a gradual decrease is observed starting from the age of 55 [2]. 

Human papillomavirus (HPV) infection is the main cause of cervical cancer. It can be spread through sexual contact. For this reason, risk factors include early initiation of sexual life and a large number of sexual partners [3,4].

Other risk factors include:

- age;
- multiparity;
- low socioeconomic status;
- unsuitable diet (diet poor in vitamin C),
- family history of cancer [5].

In May 2020, the WHO called on all global institutions involved in the prevention of cervical cancer to take action to eliminate the disease as a population-wide problem, i.e., to reduce its prevalence to that of a very rare disease (≤4 cases/100,000 subjects/year) by the end of the century. The WHO has also set shorter-term objectives with the aim to increase the likelihood of reaching the top-level goal.

The year 2030 is the deadline for the achievement of minimum goals referred to as “90-70-90”, i.e.,

- full anti-HPV vaccination of 90% of girls before the age of 15;
- high-efficiency screening tests being performed in 90% of women at least twice in their lifetime, i.e., at the age of 35 and 45;
- adequate treatment and care being provided to 90% of women with the diagnosis of precancerous lesions and cervical cancer [6].
- Primary and secondary prophylaxis are effective ways of preventing cervical cancer. Primary prevention is based primarily on immunoprophylaxis and all kinds of measures aimed at eliminating the risk factors. Secondary prevention involves screening and treatment of precancerous lesions. Screening examinations include cytology and assays for oncogenic HPV genotypes [3].

Screening programs are limited by numerous barriers. The main problem consists in low uptake as the mass scale of the programs is a prerequisite for their efficacy. Measures should be taken to promote participation in screening; in this context, the possibility of self-sampling may constitute one of the encouraging factors for potential patients [7].

## 2. Objective

The main objective of the conducted analysis was to evaluate the impact of HPV self-sampling on the efficacy of populational screening programs aimed at early detection of CC, including cost-effectiveness analysis. The study assessed the diagnostic accuracy of tests carried out in self-collected samples compared to tests carried out in samples collected by clinicians. It was also verified whether offering of HPV self-sampling kits to patients or the mailing of such kits affects the increase in the uptake of and participation in cervical cancer screening programs. In addition, it was checked whether self-sampling is accepted by women.

### Material and Method

A clinical analysis was carried out using the results of studies identified in a systematic literature review carried out according to the following outline:

- identification of reliable sources of medical information;
- searching for research papers in the full-text version that can be useful for clinical analysis;
- research selection based on inclusion criteria;
- proper processing of research results;
- qualitative synthesis of results, taking into account the analysis of the clinical and statistical significance of the research results.

The process of searching a clinical trial followed a detailed predefined protocol. The systematic review was carried out in accordance with the Cochrane Collaboration guidelines [8], specifically with regard to the inclusion criteria, search strategy, method of study selection, and analytical methodology.

Included in the analysis were clinical trials which met the following criteria:

- population: the general population of adult women;
- intervention: HPV test of a self-collected vaginal sample;
- comparator: no restriction;
- methodology: meta-analyses of randomized and/or observational studies; systematic reviews of randomized and/or observational studies; systematic reviews of cost-effectiveness analyses;
- outcomes: diagnostic accuracy (sensitivity, specificity) of CC screening tests, uptake of CC screening, CC mortality, acceptability of the sampling method, cost-effectiveness of CC screening.

The following sources of medical information were queried as part of the search for secondary research studies: Medline (via PubMed), Embase (via Ovid), and the Cochrane Library. The last search of the databases was carried out on 13 June 13 2022 in accordance with the search strategies as specified in File S1.

**Figure 1:**
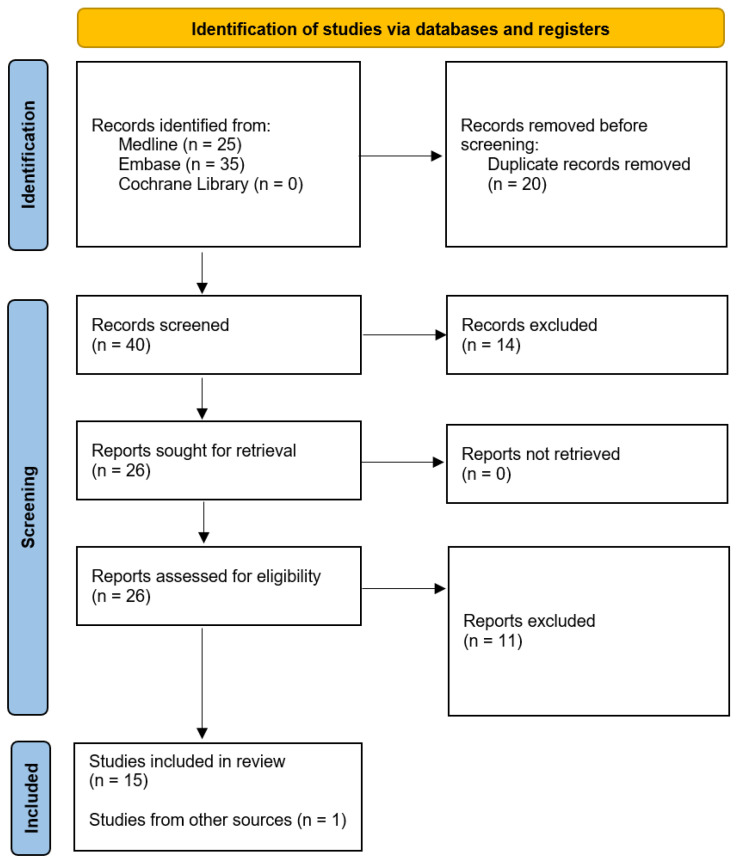
PRISMA flow diagram.

At every stage of the systematic review, the selection of studies was carried out by two independent analysts (K.W. and W.M.). All differences were resolved by consensus, with the help of a third independent analyst (J.Ś.). The most common reasons for excluding a study from the analysis were uncertainties regarding the intervention, including the lack of a detailed analysis of the HPV sampling method and methodological problems, e.g., lack of an appropriate description of the method, incorrect synthesis of the review results. The selection process is shown in Figure 1, and the list of included and excluded publications is presented as File S1. Additionally, one article selected outside the search procedure was included in the review.

The quality and risk of bias for the secondary studies included in the analysis were assessed by verifying the key domains of the AMSTAR2 systematic review appraisal tool [9]. The use of the tool facilitates the identification of top-quality publications. For a publication to obtain the highest rating, positive answers to all questions must be obtained. A single failure within a critical domain results in the review being rated as “low” quality. Two and more failures reduce this rating to “critically low”. The quality assessment was carried out by two independent analysts (K.W. and W.M.). Discrepancies were resolved by consensus with the assistance of the third independent analyst (J.Ś). For detailed results of the analysis of the quality and the risk of error of the studies, please see File S1.

The secondary studies cited the results of the statistical analyses as carried out by the authors of individual research studies (i.e., constituted reliable sources of information due to being based on original data). The results of each of the studies are presented separately.

## 3. Results 

The inclusion criteria for this systematic review were met by the following research studies: (*n* = 16; Arbyn 2022 [10], Tesfahunei 2021 [11], Malone 2020 [12], Morgan 2019 [13], Yeh 2019 [14], Arbyn 2018 [15], Kelly 2017 [16], Mezei 2017 [17], Musa 2017 [18], Nelson 2017 [19], Verdoodt 2015 [20], Albrow 2014 [21], Arbyn 2014 [22], Camilloni 2013 [23], Racey 2013 [24], Zhao 2012 [25]):

- Arbyn 2022—a meta-analysis of 26 diagnostic test accuracy studies including calculation of compatibility parameters between HPV tests in self-collected vs. clinician-collected samples [10];
- Tesfahunei 2021—a meta-analysis of 4 RCTs assessing the efficacy of HPV self-sampling as compared to standard sampling performed by clinicians at healthcare facilities for cervical cancer screening purposes [11];
- Malone 2020—a systematic review of 16 cost-effectiveness analyses assessing the use of HPV self-sampling kits as an intervention to increase uptake of CC screening programs [12];
- Morgan 2019—a systematic review of 19 cross-sectional and 4 qualitative studies aimed at identification of studies assessing the acceptability of self-sampling as compared to sampling performed by a clinician and at determination of preferences and barriers in association with both of these methods [13];
- Yeh 2019—a meta-analysis of 29 RCTs and 4 observational studies designed for the purpose of formulating the WHO guidelines and determining the impact of HPV self-sampling on the uptake of CC screening [14];
- Arbyn 2018—a meta-analysis of 81 observational studies and RCTs assessing the diagnostic accuracy of high-risk HPV (hrHPV) testing in self-sampled tests and the efficacy of the self-sampling approach on the ability of reaching out to women who have never been screened for cervical cancer [15];
- Kelly 2017—a meta-analysis of 8 observational studies comparing the diagnostic accuracy of point-of-care hrHPV tests depending on the sampling method [16];
- Mezei 2017—a systematic review of 19 cost-effectiveness analyses relating to different methods of screening for cervical cancer [17];
- Musa 2017—a meta-analysis of 28 RCTs aimed at determination of the impact of education, physicians’ recommendations to take part in the screening programs, and the availability of HPV self-sampling on the uptake of screening programs among women within the cervical cancer risk group [18];
- Nelson 2017—a meta-analysis of 37 observational and experimental studies assessing patient acceptability and preferences regarding HPV self-sampling as compared to sampling performed by clinicians [19];
- Verdoodt 2015—a meta-analysis of 16 RCTs assessing participation in CC screening following an invitation being sent along with an HPV self-sampling kit as compared to participation in following an invitation to report for a test to be performed in a clinical setting [20];
- Albrow 2014—a systematic review of 4 RCTs assessing interventions aimed at increasing the cervical cancer screening program uptake rates among women aged ≤ 35 years [21];
- Arbyn 2014—a meta-analysis of 36 observational studies and RCTs carried out to verify whether HPV self-sampling is equivalent to clinician sampling [22];
- Camilloni 2013—a meta-analysis of 69 observational and experimental studies assessing the effectiveness of interventions aimed at increasing uptake of established populational screening programs [23];
- Racey 2013—a meta-analysis of 9 RCTs and 1 observational study assessing the impact of HPV self-sampling on the increase in the participation in screening programs among women who had not previously been screened for cervical cancer [24];
- Zhao 2012—a meta-analysis of 5 populational studies comparing the diagnostic accuracy of HPV tests in self-collected samples with the accuracy of HPV tests in clinician-collected samples [25].

Below are the results of the studies returned in the query. 

### 3.1. Relationship between the Sampling Method and the Diagnostic Accuracy of HPV Test-Based Cervical Cancer Screening

In the Arbyn 2022 meta-analysis, the authors performed compatibility testing between the results of the HPV test performed in patient- vs. clinician-collected samples. The pooled test positivity ratio calculated for all 26 publications was 0.99 (95% CI: (0.94; 1.03)) suggesting no differences between the two sampling approaches regardless of the HPV testing method. The pooled overall agreement between the results of HPV tests performed in self-collected samples as compared to clinician-collected samples differed depending on whether the test was performed by means of signal amplification-based DNA assay, target amplification-based DNA assay, or an RNA assay, with the respective values amounting to 86.7% (95% CI: (0.823; 0.906)), 90.4% (95% CI: (0.874; 0.931)), and 82.3% (95% CI: (0.797; 0.847)). Regardless of the HPV testing method, the positive agreement rate between two sampling methods was 84.6% (95% CI: (0.799; 0.887)) while the negative agreement rate was 91.7% (95% CI: (0.891; 0.940)) [10].

The authors of the Arbyn 2018 paper assessed the diagnostic accuracy of tests for high-risk human papillomavirus (hrHPV) in self-collected samples against the accuracy of the test carried out in clinician-collected samples. As per the meta-analysis of 23 observational studies involving signal amplification based hrHPV assays, the relative sensitivity of CIN2+ detection within self-collected samples as compared to that within clinician-collected samples was 0.85 (95% CI: (0.80; 0.89)) whereas the relative specificity was 0.96 (95% CI: (0.93; 0.98)). On the basis of 17 studies, no statistically significant change was observed in the relative sensitivity of CIN2+ detection using PCR-based hrHPV assays depending on the sampling method, which was at the level of 0.99 (95% CI: (0.97; 1.02)). The relative specificity of CIN2+ detection was 0.98 (95% CI: (0.97; 0.99)) [15]. 

In the Kelly 2017 meta-analysis, the authors presented the diagnostic accuracy of CIN2+ and CIN3+ detection in point-of-care hrHPV assays depending on the sampling method. As shown by the meta-analysis, the sensitivity and specificity of CIN2+ detection using the self-sampled assays was 74% (95% CI: (0.65; 0.81)), and 88% (95% CI: (0.79; 0.93)), respectively, while the sensitivity of 88% (95% CI: (0.81; 0.93))), and the specificity of 84% (95% CI: (0.75; 0.90)) were calculated for the same assays being carried out in clinician-collected samples. With regard to CIN3+ detection, the sensitivity and specificity of the self-sampled hrHPV test were 75% (95% CI: (0.67; 0.82)), and 91% (95% CI: (0.83; 0.95)), respectively, while the clinicians-sampled test was characterized by the sensitivity of 90% (95% CI: (0.84; 0.94))), and the specificity of 85% (95% CI: (0.73; 0.92)) [16].

In the Arbyn 2014 meta-analysis, the authors assessed whether the HPV tests in self-collected samples were equivalent to HPV tests in clinician-collected samples. As shown by the results pooled from 14 RCTs, the sensitivity and specificity of the CIN2+ detection in the HPV assay carried out in a patient-collected sample were 76% (95% CI: (0.69; 0.82)), and 86% (95% CI: (0.83; 0.89)), respectively. The sensitivity and specificity of HPV assays related to primary screening carried out in clinician-collected samples were 91% (95% CI: (0.87; 0.94)), and 88% (95% CI: (0.85; 0.91)), respectively [22]. The Zhao 2012 meta-analysis presented the results of the sensitivity and specificity of HPV tests based on 5 populational cervical cancer screening programs in China. As part of the programs, HC2 tests were performed in self-collected and clinician-collected samples. As shown by the results, the sensitivity and specificity of CIN2+ detection in self-sampled assays amounted to 86.2% (95% CI: (0.829; 0.891)), and 80.7% (95% CI: (0.756; 0.858)), respectively. The sensitivity and specificity of CIN3+ detection amounted to 86.1% (95% CI: (0.814; 0.90)), and 79.5% (95% CI: (0.741; 0.848)), respectively. The sensitivity and specificity of CIN2+ detection in clinician-sampled HPV assays amounted to 97% (95% CI: (0.952; 0.983)), and 82.7% (95% CI: (0.784; 0.870)), respectively. The sensitivity and specificity of CIN3+ detection in clinician-sampled HPV assays amounted to 97.8% (95% CI: (0.953; 0.992)), and 81.3% (95% CI: (0.767; 0.858)), respectively [25]. 

Presented below are the characteristics and diagnostic accuracy of HPV tests from self-collected samples and clinician-collected samples (Table 1).

### 3.2. Uptake of CC Screening

The authors of the Yeh 2019 meta-analysis assessed different methods used to deliver HPV self-sampling kits to women eligible for screening yet having never taken a test. On the basis of a total of 29 RCTs, the meta-analysis showed that mailing of the HPV sampling kits significantly improved uptake of screening as compared to invitations for a screening examination to be carried out by a clinician (cytology, visual inspection with acetic acid (VIA), or HPV): RR = 2.13 (95% CI: (1.89; 2.40)). In addition, with regard to the sampling kit distribution strategy, a statistically significant, more-than-twofold increase in uptake was achieved when kits were delivered by mail (RR = 2.27 (95% CI: (1.89; 2.71))) or by a health worker (door-to-door strategy) (RR = 2.37 (95% CI: (1.12; 5.03))) [14].

Two meta-analyses (Arbyn 2018 and Verdodt 2015) compared the methods of delivery of HPV self-sampling kits to women who did not do regular screening tests or women who had never had a screening test in the past. In both meta-analyses, mailing the HPV self-sampling kits was shown to result in statistically significant, more-than twofold increases in uptake as compared to the invitation for or reminder about a screening test to be performed by a clinician (RR = 2.33 (95% CI: (1.86; 2.91)) [15]; RR = 2.40 (95% CI: (1.73; 3.33))) [20]. In addition, offering HPV self-screening kits to women as part of a social campaign had a statistically significant impact consisting in uptake being increased by a factor of more than 2.5 (as compared to handing out invitations for cytology tests, RR = 2.58 (95% CI (1.67; 3.99)) [15]. Neither of the meta-analyses could demonstrate a statistically significant impact of the opt-in strategy (confirmation of willingness to participate in the study) or the door-to-door strategy (kits delivered and received by another person, e.g., nurse, community health worker) on the uptake rate [15,20].

Another meta-analysis, carried out by Camilloni in 2013 and based on 7 observational studies, compared the effectiveness of HPV self-sampling kits being distributed by mail to that of reminders about a cytological exam to be performed at a health center. Mailing self-sampling kits to women eligible for CC screening who had not reported to a cytological exam was shown to statistically significantly increase screening uptake (RR = 2.37 (95% CI: (1.44; 3.90))) [23].

The Racey 2013 meta-analysis showed that distribution of HPV self-sampling kits (by mail or by means of the door-to-door strategy) to female patients living in developed countries who had never taken part in CC screening resulted in a more-than-twofold increase in uptake as compared to invitations for cytology exams: RR = 2.14 (95% CI: (1.30; 3.52)) [24]. As highlighted by the authors of the Racey 2013 report, the presented results were highly heterogeneous.

As shown in one of the meta-analyses included in this review (Tesfahunei 2021), the availability of self-sampling had an overall statistically significant effect on increased uptake of CC screening in the population of women living in sub-Saharan Africa: RR = 1.72 (95% CI: (1.58; 1.87)) (4 RCTs). To account for the heterogeneity of the studies included in this meta-analysis, the authors performed their analysis in two subgroups divided on the basis of time to sample delivery (1—women were allowed to return the kit within a certain time frame; 2—women were asked to perform self-sampling immediately after receiving the offer). One RCT showed that the uptake of screening was significantly higher in the group of subjects who collected the samples immediately upon being offered to undergo screening: RR = 2.05 (95% CI: (1.80; 2.33)). Allowing the patients to perform the test within a certain time frame was also associated with a significant increase in the uptake rate although the effect was lower than in the first subgroup: RR = 1.65 (95% CI: (1.58; 1.72)) (3 RCTs). A statistically significant difference between both subgroups was revealed in the analysis (*p* = 0.001) [11].

As demonstrated by the Musa 2017 meta-analysis of 8 RCTs, self-sampling in women eligible for CC screening significantly increased the uptake rate by 71% (RR = 1.71 (95% CI: (1.32; 2.22))) [18]. Similar results were obtained in the systematic review of Albrow 2014, where a statistically significant increase of 23% was observed for the uptake rate (RR = 1.23 (95% CI: (1.01; 1.48))) [21].

Presented below are the characteristics and results of individual CC screening reportability studies (Table 2).

### 3.3. The Influence of Self-Sampling on CC Detection and Mortality Rates

No secondary scientific reports relating to the effects of HPV self-sampling on CC detection and mortality rates were found in a systematic review of available literature.

### 3.4. The Acceptability of Self-Sampling among Patients 

The Nelson 2017 meta-analysis assessed patient acceptability and preferences regarding HPV self-sampling as compared to sampling performed by clinicians. According to the results (7 studies, N = 1470), 97% of patients (95% CI: (0.95; 0.98)) declared self-sampling to be generally acceptable. In addition, according to a meta-analysis of 9 studies, 87% of women (95% CI: (0.73; 0.95)) would be willing to repeat self-sampling in the future. According to a meta-analysis of 23 studies involving a total of 12,610 women, the average self-sampling preference rate was 59% (95% CI: (0.48; 0.69)) [19].

The authors of the Nelson 2017 meta-analysis had listed the reasons behind patients’ willingness to perform self-sampling rather than have a sample collected by a clinician as reported in a total of 34 studies. The most common reasons behind the preference for self-sampling included the ease of use (91% (95% CI: (0.86; 0.94))), lack of the feeling of embarrassment (91% (95% CI: (0.83; 0.95))), privacy (88% (95% CI: (0.65; 0.97))), comfort (88% (95% CI: (0.82–0.91))), ability to self-perform the test (69% (95% CI: (0.46; 0.85))), and convenience (65% (95% CI: (0.42; 0.83))). Reasons for the refusal or unwillingness to perform a self-sampled HPV test were also listed by the authors. According to the presented data, the most common causes included uncertainty about the correct performance of the self-sampling procedure (21% (95% CI: (0.14; 0.32))), anxiety (15% (95% CI: (0.07; 0.23))), tenderness of physical discomfort (10% (95% CI: (0.06; 0.17))), and unwillingness to touch one’s private parts (6% (95% CI: (0.02; 0.19))) [19].

Likewise, the Morgan 2019 systematic review focused on barriers and preferences regarding to HPV self-sampling as compared to sampling performed by clinicians. On the basis of 8 cross-sectional studies, self-sampling was found to be considered easy to perform by 69–95% of patients. In 5 studies, patients preferred self-sampling because of the greater privacy as compared to the sampling performed by a clinician [13]. In 10 of the studies included in the review, the responders considered the sampling performed by the clinician and the result of the test based thereon to be more reliable than in the self-sampling scenario. As shown by the results of 3 studies, 56.7–87.2% of patients would be willing to repeat self-sampling in the future.

### 3.5. Cost-Effectiveness Analyses

The authors of the Malone 2020 systematic review attempted to estimate the cost-effectiveness of HPV self-sampling-based screening as an intervention leading to increased CC screening uptake rates. The analysis was carried out in the overall population of women. Depending on the source publication, the intervals between successive tests ranged between 3 and 10 years. The cost-effectiveness thresholds reported in the systematic review were diverse and ranged from $756/LYG (Uganda) to $136,672/QALY (Norway). A standard invitation for a cytology exam was used as a comparator in the studies included in the review. As shown by the cost-effectiveness analyses, mailing HPV self-sampling kits every three years was found to be cost-effective in the group of female subjects aged 25–70, with the incremental cost-effectiveness ratio (ICER) amounting to $10,898/QALY as compared to the pre-determined cost-effectiveness threshold of $48,922/QALY. With regard to the remaining strategies taken into account in the systematic review, most of these strategies were also found to be cost-effective. In some cases, the assessed strategies were dominated by the comparator. The obtained ICER values depended on the target population, intervals between consecutive tests, and the target screening method (including further follow-up) as chosen by the payer. Based on an extensive narrative analysis, the authors concluded that HPV self-sampling can be cost-effective in the context of increasing the cervical cancer screening uptake rates. Thus, Malone et al. indicate that self-sampling can be a cost-effective method to increase cervical cancer screening uptake and prevent cervical cancer under certain settings and conditions [12].

The authors of the Mezei 2017 systematic review assessed the cost-effectiveness of different CC screening strategies. The reported HPV test-based strategies involved tests being performed once in a lifetime at an age of 35 and the screening process consisting of two visits (screening + results and treatment if positive) while varying in terms of the HPV sampling method (self- vs. clinician-sampled). The ICER was expressed in terms of cost per year of life saved (YLS). According to the results of the studies included in this review, the ICER for the screening based on HPV self-sampling ranged from I$1800/YLS to $2020/YLS, with the strategy of interest being dominated by a single visit involving a VIA exam in two of the studies (costs were standardized to 2005 value rates of US dollars and international dollars (I$)). The ICER for clinician-sampled HPV tests ranged from $50/YLS to $2040/YLS; in three cases, VIA as performed during a single clinical visit dominated over the screening strategy of interest. The average direct medical costs of performing the screening tests of interest were also presented by the authors of the review. According to the presented results, the average cost of a self-sampled HPV test amounted to $7.50 (2005 value rate) and $12.28 I$ (2005 value rate). The average cost of a clinician-sampled HPV assay was $13.27 (2005 value rate) and $17.72 (2005 value rate). According to the information provided in the review, the provider-collected HPV tests were generally more effective than self-sampled with the exception of scenarios assuming that self-sampling would increase the populational coverage [17].

## 4. Discussion

The sensitivity and specificity of HPV self-sampling were assessed on the basis of the results of studies returned in the query. Publications on the patients’ acceptability of self-sampling and the impact of this method on CC screening uptake rates were also analyzed.

The HPV assays performed in self-collected samples are characterized by sensitivity and specificity values being acceptable but lower than in the case of HPV assays in clinician-collected samples [15,22,25]. However, the slight reduction in these parameters is compensated by a much higher (even more-than-twofold) subjects’ willingness to participate in the self-sampled screening as compared to assays performed at the clinic (HPV or cytology tests) [11,14,15,20,23,24]. Self-sampling is also widely acceptable for subjects [13,19].

The manner in which female subjects are invited and provided with self-sampling kits is also important. According to the obtained results, the opt-in approach is not a good way to increase participation in screening [13,14,19]. The best results were obtained when the self-sampling kits were delivered by mail [14,15,20,23]. A positive impact on the uptake rate was also observed following social campaigns within in local communities and mass media [15]. 

No secondary scientific reports meeting the inclusion criteria and relating to the effects of HPV self-sampling on CC detection and mortality rates were found in a systematic review of available literature. Therefore, these parameters were not analyzed in this publication.

For the purpose of this discussion, a review was made of the current clinical practice guidelines for CC screening with a particular focus on HPV self-sampling. 

The authors of the recommendations agree that the main means to prevent the consequences of cervical cancer consist in early detection screening. The screening methods as most frequently reported by scientific societies include cytology [26,27,28,29,30,31,32,33,34,35] and HPV assays [26,27,28,29,30,31,33,36,37,38,39,40]. A co-testing option in which both methods are used has also been mentioned in the recommendations [26,27,28,29,30,31,32,41].

As highlighted in the identified recommendations from scientific societies, the CC screening programs should focus mainly on the population of young women. Numerous guidelines recommend regular screening starting from the age of 21 [26,31,32,36,41] or 25 [27,28,30,35,38,39,40]. Most guidelines suggest discontinuation of screening in women above the age of 65 years after negative results have been obtained in the previous screening [26,27,28,30,31,32,33,36,37,38,41]. In some guidelines, the upper age limit is set at 69 [35] or even 74 years [39,40]. 

Recommendations are not unanimous with regard to the age at which initiation of HPV testing is recommended. According to some documents, HPV testing is recommended in women aged 30 or older [26,28,30,32] whereas other studies suggest that tests can be done starting from the age of 25 [27,36,39,40].

Below are the recommendations from different institutions regarding HPV self-sampling. Some recommendations are clearly in favor of the self-sampling approach whereas others point to the lack of sufficient evidence allowing for unconditional application of this method.

The American Cancer Society (ACS) strongly recommends that HPV tests are performed in patient-collected samples [27]. 

According to the WHO, screening programs may be based on HPV tests performed in patient- as well as in clinician-collected samples [37].

The United States Preventive Services Task Force (USPSTF) declares that HPV samples can be self-collected by patients (in contrast to cytology samples) and returned for examination. According to USPSTF, this strategy may be useful for increasing the uptake rates for screening programs, although “rigorous comparative studies [are required] to verify this hypothesis and to identify effective strategies for implementation” [30].

In turn, the American College of Obstetricians and Gynecologists (ACOG) state that despite the increasing evidence base, self-sampling is still in the exploratory phase [26]. 

A report by the European Commission (EC) states that “the clinical accuracy of HPV primary testing on self-collected samples taken for cervical screening is sufficient to conduct organized, population-based pilot programs for women who have not attended screening despite a personal invitation and a personal reminder” [33]. The National Cancer Institute (NCI) remarks that self-sampling may be an alternative method for use in screening programs, particularly in communities with limited access to healthcare providers [41].

The European Society of Gynaecologic Oncology (ESGO) and the European Federation of Colposcopy (EFC) point out that self-sampling kits may only be sent to women who did not respond to invitations to participate in a conventional clinician-sampled test. It is highlighted that sending kits to all women (i.e., not only to those who have not responded to invitations) is not recommended [38]. A different position has been included in the recommendations of the Cancer Council Australia (CCA) [39]. According to this document, it is acceptable and recommended to offer self-sampling kits to all women eligible for screening (aged 25 to 74 years as per the institutional recommendations).

Considering all of the above, it seems that HPV self-sampling is set to be (and sometimes has already become) the main method of conducting populational tests. An increasing number of scientific reports point to the effectiveness of this type of screening, particularly considering the greater willingness to participate among potential patients.

In accordance with the adopted methodology, all studies included in the systematic review concerned self-collection of samples for the HPV test by vaginal self-sampling. However, it should be remembered that there is also a method that allows the analysis of urine samples.

The Cho 2019 study found for discussion indicates that HPV tests using self-collected vaginal and urine samples show significant and moderate agreement, respectively, compared to clinician-collected cervical samples. Further studies are needed on the clinical effectiveness of HPV tests using urine and self-collected vaginal samples as screening methods [42]. The authors of the Cadman 2021 study on the comparison of different methods of self-collection of vaginal and urine samples for HPV testing drew attention to women’s preferences in this respect, indicating that urine collection was considered by the study participants as the easiest and gave them the greatest certainty that they had taken the sample correctly [43]. Testing urine samples is therefore a promising method of self-sampling, but requires further research, in particular in the field of test accuracy [44,45].

## 5. Limitations of the Review

Only English-language papers were included in the query. The search was restricted to reports published within the last 10 years (13 June 2012–13 June 2022). Studies included as secondary evidence were carried out in ethnically and geographically diverse populations. In addition, the studies themselves were characterized by significant heterogeneity and different methods being used to present the analyzed data. One should also keep in mind that the returned studies had been performed within the specific cultural, economic, and organizational contexts of the healthcare systems in the countries of origin. A significant limitation of this review consists in the lack of secondary scientific reports meeting the inclusion criteria and relating to the effects of HPV self-sampling on the CC detection and mortality rates which prevented the relevant analyses.

## 6. Conclusions

Cervical cancer is a serious public health problem. Through its classic symptoms, it significantly reduces the quality of life and patients’ life expectancy. Timely detection of an early stage of cancer facilitates treatment and increases the chance of being cured. Self-sampling for HPV is an innovative and cost-effective way to perform screening and has a significant impact on the willingness to participate in screening programs among the female population.

Most of the identified studies indicate that HPV self-sampling is acceptable for the subjects. The highest uptake rates were achieved when sampling kits were sent to the subjects by mail. On the other hand, the opt-in strategy failed to increase the uptake rate and should therefore not be widely used.

## Figures and Tables

**Table 1:** Characteristics and diagnostic accuracy of HPV tests from self-collected samples.

Author/Year	Population	HPV Testing Method	End Point	Accuracy of CIN2+ Detection		Accuracy of CIN3+ Detection	
				Sensitivity (95% CI) (*n* Studies)	Specificity (95% CI) (*n* Studies)	Sensitivity (95% CI) (*n* Studies)	Specificity (95% CI) (*n* Studies)
Arbyn 2018 [15] (MA)	Women who did not do regular screening tests or women who had never had a screening test in the past	hrHPV assays based on signal amplification	Accuracy of the HPV test in self-collected samples as compared to that performed in clinician-collected samples	R = 0.85 (0.80–0.89) (23 OS)	R = 0.96 (0.93–0.98) (23 OS)	R = 0.86 (0.76–0.98) (9 OS)	R = 0.97 (0.95–0.99) (9 OS)
		hrHPV assays based on PCR		R = 0.99 (0.97–1.02) (17 OS)	R = 0.98 (0.97–0.99) (17 OS)	R = 0.99 (0.96–1.02) (8 OS)	R = 0.98 (0.97–0.99) (8 OS)
Kelly 2017 [16] (MA)	Sexually active women (including women with HIV infection) included in the screening program	hrHPV assays based on signal amplification	Accuracy of the HPV test in self-collected samples	74% (0.65–0.81) (4 OS)	88% (0.79–0.93) (4 OS)	75% (0.67–0.82) (3 OS)	91% (0.83–0.95) (3 OS)
		hrHPV assays based on signal amplification	Accuracy of the HPV test in clinician-collected samples	88% (0.81; 0.93) (7 OS)	84% (0.75; 0.90) (7 OS)	90% (0.84; 0.94) (4 OS)	85% (0.73; 0.92) (4 OS)
Arbyn 2014 [22] (MA)	Women participating in a screening program	-	Absolute accuracy of the HPV test in self-collected samples	76% (0.69–0.82) (14 RCT)	86% (0.83–0.89) (14 RCT)	84% (0.72–0.92) (6 RCT)	87% (0.84–0.90) (6 RCT)
		-	Absolute accuracy of the HPV test in clinician-collected samples	91% (0.87–0.94) (14 RCT)	88% (0.85–0.91) (14 RCT)	95% (0.91–0.97) (6 RCT)	89% (0.87–0.92) (6 RCT)
	Women in high-risk groups	-	Absolute accuracy of the HPV test in self-collected samples	75% (0.58–0.87) (3 RCT)	86% (0.77–0.92) (3 RCT)	42% (0.27–0.57) (1 RCT)	81% (0.76–0.87) (1 RCT)
		-	Absolute accuracy of the HPV test in clinician-collected samples	88% (0.78–0.93) (3 RCT)	88% (0.81–0.93) (3 RCT)	80% (0.67–0.93) (1 RCT)	82% (0.77–0.88) (1 RCT)
Zhao 2012 [25] (MA)	Women aged 17–56 years participating in the populational screening program	Hybrid Capture 2 assay	Accuracy of the HPV test in self-collected samples	86.2% (0.829–0.891) (5 OS)	80.7% (0.756–0.858) (5 OS)	86.1% (0.814–0.90) (5 OS)	79.5% (0.741–0.848) (5 OS)
	Women aged 17–56 years participating in the populational screening program	Hybrid Capture 2 assay	Accuracy of the HPV test in clinician-collected samples	97% (0.952–0.983) (5 OS)	82.7% (0.784–0.870) (5 OS)	97.8% (0.953–0.992) (5 OS)	81.3% (0.767–0.858) (5 OS)

CIN—cervical intraepithelial neoplasia; HPV—human papillomavirus; hrHPV—high-risk human papillomavirus; OS—observational study; R—ratio; RCT—randomized controlled trial.

**Table 2:** Characteristics and results of CC screening reportability studies.

Author/Year	N of Studies	Population		Intervention		Comparator	End Point	RR (95% CI)
		Population Summary	Population Size (*n*/N) *					
Tesfahunei [11] 2021 (MA)	4 RCTs	Female residents of sub-Saharan Africa, aged 25–65	3192/4561 (I) 1566/3639 (C)	HPV self-sampling offer	Overall	Screening to be performed by a clinician at a health care facility (HPV test or VIA)	Screening uptake	RR = 1.72 (1.58–1.87)
	3 RCTs		2944/4311(I) 1445/3389 (C)		Within some time range			RR = 1.65 (1.58–1.72)
	1 RCT		248/250 (I) 121/250 (C)		Immediately on recruitment			RR = 2.05 (1.80–2.33)
Yeh 2019 [14] (MA)	29 RCTs	Women eligible for CC screening who had not reported for a cytology test.	307,960	HPV self-sampling kit delivery	Overall	Invitation for a screening test to be performed by a clinician (cytology, VIA, HPV assay)	Screening uptake	RR = 2.13 (1.89–2.40)
	23 RCTs		276,229		By mail			RR = 2.27 (1.89–2.71)
	5 RCTs		88,222		Opt-in strategy			RR = 1.28 (0.90–1.82)
	5 RCTs		32,238		Door-to-door strategy			RR = 2.37 (1.12–5.03)
Arbyn 2018 [15] (MA)	19 RCTs	Women who did not do regular screening tests or women who had never had a screening test in the past.	Not specified	HPV self-sampling kit delivery	By mail	Invitation for or reminder about a screening test to be performed by a clinician	Participation	RR = 2.33 (1.86–2.91)
	6 RCTs				Opt-in strategy			RR = 1.22 (0.93–1.61)
	4 RCTs				Door-to-door strategy			RR = 2.01 (0.66–6.15)
	1 RCT			Social campaign				RR = 2.58 (1.67–3.99)
Musa 2017 [18] (MA)	8 RCTs	Women eligible for CC screening	6154/22,256 (I) 5181/18,314 (C)	HPV self-sampling offer		Screening reminder sent by mail	Screening uptake	RR = 1.71 (1.32–2.22)
Verdoodt [20] 2015 (MA)	13 RCTs	Women who did not do regular screening tests or women who had never had a screening test in the past.	90,191 (I) 39,253 (C)	HPV self-sampling kit delivery	By mail	Invitation for or reminder about a screening test to be performed by a clinician	Participation	RR = 2.40 (1.73–3.33)
	3 RCTs		11,067 (I) 10,247 (C)		Opt-in strategy			RR = 0.97 (0.65–1.46)
	2 RCTs		12,420 (I) 16,749 (C)		Door-to-door strategy			RR = 2.21 (0.32–15.48)
Albrow 2014 [21] (SR)	1 RCT	Women eligible for CC screening	110/413 (I) 246/1114 (C)	HPV self-sampling offer		Screening reminder sent by mail	Screening uptake	RR = 1.23 (1.01–1.48)
Camilloni [23] 2013 (MA)	7 observational studies	Women aged 25–64 and eligible for CC screening who had not reported for a cytology test.	3357/64,256 (I) 1150/34,496 (C)	HPV self-sampling kit delivered by mail		A reminder regarding cytological examination to be performed at a health center	Screening uptake	RR = 2.37 (1.44–3.90)
Racey 2013 [24] (MA)	9 RCTs 1 observational study	Women in developed countries who had never had a screening test in the past.	28,143/74,312 (I) 13,466/28,369 (C)	HPV self-sampling kit delivered by mail or using the door-to-door strategy		Invitation for a screening test to be performed by a clinician (cytology)	Participation	RR = 2.14 (1.30–3.52)

* *n* = case; N = number of people in the intervention or control group. (I)—interventional group; (C)—control group; CC—cervical cancer; MA—meta-analysis; SR—systematic review; CI—confidence interval; HPV—human papillomavirus; RCT—randomized controlled trial; RR—risk ratio; VIA—visual inspection with acetic acid.

## Supplementary Materials

The following supporting information can be downloaded at: https://www.mdpi.com/article/10.3390/cancers14235913/s1, File S1: Search strategy.
