# Supplementary material for: The Influence of Vaginal HPV Self-Sampling on the Efficacy of Populational Screening for Cervical Cancer—An Umbrella Review
Source: Cancers (Basel). 2022 Nov 30;14(23):5913. doi: 10.3390/cancers14235913 (PMC9740498; doi:10.3390/cancers14235913)
Supplement: Supplementary file 1 [file cancers-14-05913-s001.zip › cancers-2035623-supplementary.pdf]

Search strategy Cochrane

ID	Keyword	Result
#1	MeSH descriptor: [Uterine Cervical Neoplasms] explode all trees	2295
#2	(cervical cancer):ti,ab,kw	5352
#3	(cervical neoplasm*):ti,ab,kw	3067
#4	(cervix cancer):ti,ab,kw	2909
#5	(cervix neoplasm*):ti,ab,kw	1329
#6	#1 OR #2 OR #3 OR #4 OR #5	6866
#7	(self-sampling):ti,ab,kw	236
#8	(selfsampling):ti,ab,kw	228
#9	(self-collected):ti,ab,kw	222
#10	(selfcollected):ti,ab,kw	212
#11	#7 OR #8 OR #9 OR #10	410
#12	MeSH descriptor: [Mass Screening] explode all trees	4112
#13	(screen*):ti,ab,kw	84503
#14	(early diagno*):ti,ab,kw	29451
#15	(early test*):ti,ab,kw	34051
#16	(early detect*):ti,ab,kw	14100
#17	#12 OR #13 OR #14 OR #15 OR #16	135008
#18	MeSH descriptor: [Alphapapillomavirus] explode all trees	282
#19	(Human Papillomavirus):ti,ab,kw	2390
#20	(HPV):ti,ab,kw	3283
#21	MeSH descriptor: [Human Papillomavirus DNA Tests] explode all trees	10
#22	(Human Papillomavirus DNA Tests):ti,ab,kw	106
#23	#18 OR #19 OR #20 OR #21 OR #22	3668
#24	#11 AND #17 AND #23	231
#25	#6 AND #24	211
#26	#6 AND #24 with Cochrane Library publication date Between Jun 2012 and Jun 2022, in Cochrane Reviews	0

Search strategy Medline (via PubMed)

ID	Keyword	Result
#1	Search: Cervical Cancer[MeSH Terms]	81965
#2	Search: cervical cancer[Title/Abstract]	54797
#3	Search: cervical neoplasm*[Title/Abstract]	1202
#4	Search: cervix cancer[Title/Abstract]	2267
#5	Search: cervix neoplasm*[Title/Abstract]	4285

#6	Search: ((((Cervical Cancer[MeSH Terms]) OR (cervical cancer[Title/Abstract])) OR (cervical neoplasm*[Title/Abstract])) OR (cervix cancer[Title/Abstract])) OR (cervix neoplasm*[Title/Abstract])	99662
#7	Search: self-sampling[Title/Abstract]	881
#8	Search: selfsampling[Title/Abstract]	825
#9	Search: self-collected[Title/Abstract]	1302
#10	Search: selfcollected[Title/Abstract]	1271
#11	Search: (((self-sampling[Title/Abstract]) OR (selfsampling[Title/Abstract])) OR (self-collected[Title/Abstract])) OR (selfcollected[Title/Abstract])	1990
#12	Search: Screening[MeSH Terms]	166175
#13	Search: screen*[Title/Abstract]	890905
#14	Search: early diagno*[Title/Abstract]	100237
#15	Search: early test*[Title/Abstract]	753
#16	Search: early detect*[Title/Abstract]	75682
#17	Search: ((((Screening[MeSH Terms]) OR (screen*[Title/Abstract])) OR (early diagno*[Title/Abstract])) OR (early test*[Title/Abstract])) OR (early detect*[Title/Abstract])	1081495
#18	Search: Human Papillomavirus[MeSH Terms]	9528
#19	Search: Human Papillomavirus[Title/Abstract]	40004
#20	Search: HPV[Title/Abstract]	48146
#21	Search: Human Papillomavirus DNA Tests[MeSH Terms]	568
#22	Search: Human Papillomavirus DNA Tests[Title/Abstract]	62
#23	Search: ((((Human Papillomavirus[MeSH Terms]) OR (Human Papillomavirus[Title/Abstract])) OR (HPV[Title/Abstract])) OR (Human Papillomavirus DNA Tests[MeSH Terms])) OR (Human Papillomavirus DNA Tests[Title/Abstract])	57319
#24	Search: (((((self-sampling[Title/Abstract]) OR (selfsampling[Title/Abstract])) OR (self-collected[Title/Abstract])) OR (selfcollected[Title/Abstract])) AND (((((Screening[MeSH Terms]) OR (screen*[Title/Abstract])) OR (early diagno*[Title/Abstract])) OR (early test*[Title/Abstract])) OR (early detect*[Title/Abstract]))) AND (((((Human Papillomavirus[MeSH Terms]) OR (Human Papillomavirus[Title/Abstract])) OR (HPV[Title/Abstract])) OR (Human Papillomavirus DNA Tests[MeSH Terms])) OR (Human Papillomavirus DNA Tests[Title/Abstract]))	750
#25	Search: (((((Cervical Cancer[MeSH Terms]) OR (cervical cancer[Title/Abstract])) OR (cervical neoplasm*[Title/Abstract])) OR (cervix cancer[Title/Abstract])) OR (cervix neoplasm*[Title/Abstract])) AND ((((((self-sampling[Title/Abstract]) OR (selfsampling[Title/Abstract])) OR (self-collected[Title/Abstract])) OR (selfcollected[Title/Abstract])) AND (((((Screening[MeSH Terms]) OR (screen*[Title/Abstract])) OR (early diagno*[Title/Abstract])) OR (early test*[Title/Abstract])) OR (early detect*[Title/Abstract]))) AND	692

	(((((Human Papillomavirus[MeSH Terms]) OR (Human Papillomavirus[Title/Abstract])) OR (HPV[Title/Abstract])) OR (Human Papillomavirus DNA Tests[MeSH Terms])) OR (Human Papillomavirus DNA Tests[Title/Abstract])))	
#26	Search: (((((((systematic[Title]) AND ((Review[Title/Abstract]) OR "Review" [Publication Type])))))))	196489
#27	Search: ((((((((metaanalysis[Title/Abstract]) OR Meta-Analysis[Title/Abstract]) OR "Meta-Analysis" [Publication Type]))))))	235534
#28	Search: ((((((((systematic[Title]) AND ((Review[Title/Abstract]) OR "Review" [Publication Type])))))))) OR (((((((((metaanalysis[Title/Abstract]) OR Meta-Analysis[Title/Abstract]) OR "Meta-Analysis" [Publication Type])))))))	337718
#29	Search: ((((((Cervical Cancer[MeSH Terms]) OR (cervical cancer[Title/Abstract])) OR (cervical neoplasm*[Title/Abstract])) OR (cervix cancer[Title/Abstract])) OR (cervix neoplasm*[Title/Abstract])) AND ((((((self-sampling[Title/Abstract]) OR (selfsampling[Title/Abstract])) OR (self-collected[Title/Abstract])) OR (selfcollected[Title/Abstract])) AND (((((Screening[MeSH Terms]) OR (screen*[Title/Abstract])) OR (early diagno*[Title/Abstract])) OR (early test*[Title/Abstract])) OR (early detect*[Title/Abstract]))) AND (((((Human Papillomavirus[MeSH Terms]) OR (Human Papillomavirus[Title/Abstract])) OR (HPV[Title/Abstract])) OR (Human Papillomavirus DNA Tests[MeSH Terms])) OR (Human Papillomavirus DNA Tests[Title/Abstract])))) AND (((((((((systematic[Title]) AND ((Review[Title/Abstract]) OR "Review" [Publication Type])))))))) OR (((((((((metaanalysis[Title/Abstract]) OR Meta-Analysis[Title/Abstract]) OR "Meta-Analysis" [Publication Type]))))))))	28
#30	Search: ((((((Cervical Cancer[MeSH Terms]) OR (cervical cancer[Title/Abstract])) OR (cervical neoplasm*[Title/Abstract])) OR (cervix cancer[Title/Abstract])) OR (cervix neoplasm*[Title/Abstract])) AND ((((((self-sampling[Title/Abstract]) OR (selfsampling[Title/Abstract])) OR (self-collected[Title/Abstract])) OR (selfcollected[Title/Abstract])) AND (((((Screening[MeSH Terms]) OR (screen*[Title/Abstract])) OR (early diagno*[Title/Abstract])) OR (early test*[Title/Abstract])) OR (early detect*[Title/Abstract]))) AND (((((Human Papillomavirus[MeSH Terms]) OR (Human Papillomavirus[Title/Abstract])) OR (HPV[Title/Abstract])) OR (Human Papillomavirus DNA Tests[MeSH Terms])) OR (Human Papillomavirus DNA Tests[Title/Abstract])))) AND (((((((((systematic[Title]) AND ((Review[Title/Abstract]) OR "Review" [Publication Type])))))))) OR (((((((((metaanalysis[Title/Abstract]) OR Meta-Analysis[Title/Abstract]) OR "Meta-Analysis" [Publication Type])))))))) Filters: in the last 10 years	25

Search strategy Embase (via Ovid)

ID	Keyword	Result
1	exp uterine cervix cancer/	102948
2	cervical cancer.ab,kw,ti.	73669
3	"cervical neoplasm*".ab,kw,ti.	835
4	cervix cancer.ab,kw,ti.	3064
5	"cervix neoplasm*".ab,kw,ti.	771
6	1 or 2 or 3 or 4 or 5	120009
7	self-sampling.ab,kw,ti.	1124
8	selfsampling.ab,kw,ti.	61
9	self-collected.ab,kw,ti.	1704
10	selfcollected.ab,kw,ti.	60
11	7 or 8 or 9 or 10	2636
12	exp screening/	699331
13	"screen*".ab,kw,ti.	1194814
14	"early diagno*".ab,kw,ti.	132660
15	"early test*".ab,kw,ti.	943
16	"early detect*".ab,kw,ti.	105068
17	12 or 13 or 14 or 15 or 16	1644420
18	exp Wart virus/	40097
19	Human Papillomavirus.ab,kw,ti.	47183
20	HPV.ab,kw,ti.	65017
21	exp Human papillomavirus DNA test/	2420
22	Human Papillomavirus DNA Tests.ab,kw,ti.	81
23	18 or 19 or 20 or 21 or 22	82723
24	6 and 11 and 17 and 23	833
25	limit 24 to ((consensus development or meta analysis or "systematic review") and last 10 years)	35

List of studies included and excluded after full-text analysis

Lp.	Authors, Title, Journal	Full text status
1	Malone, C.; Barnabas, R.V.; Buist, D.S.M.; Tiro, J.A.; Winer, R.L. Cost-effectiveness studies of HPV self-sampling: A systematic review. Prev Med. 2020, 132, 105953.	Included
2	Arbyn, M.; Smith, S.B.; Temin, S.; Sultana, F.; Castle, P.; Collaboration on Self-Sampling and HPV Testing. Detecting cervical precancer and reaching underscreened women by using HPV testing on self samples: updated meta-analyses. BMJ 2018, 363, k4823.	Included
3	Musa, J.; Achenbach, C.J.; O'Dwyer, L.C.; Evans, C.T.; McHugh, M.; Hou, L.; Simon, M.A.; Murphy, R.L.; Jordan, N. Effect of cervical cancer education and provider recommendation for screening on screening rates: A systematic review and meta-analysis. PLoS One 2017, 12(9), e0183924.	Included
4	Nelson, E.J.; Maynard, B.R.; Loux, T.; Fatla, J.; Gordon, R.; Arnold, L.D. The acceptability of self-sampled screening for HPV DNA: a systematic review and meta-analysis. Sex. Transm. Infect. 2017, 93(1), 56-61.	Included
5	Verdoodt, F.; Jentschke, M.; Hillemanns, P.; Racey, C.S.; Snijders, P.J.; Arbyn, M. Reaching women who do not participate in the regular cervical cancer screening programme by offering self-sampling kits: a systematic review and meta-analysis of randomised trials. Eur. J. Cancer 2015, 51(16), 2375-85.	Included
6	Albrow, R.; Blomberg, K.; Kitchener, H.; Brabin, L.; Patnick, J.; Tishelman, C.; Törnberg, S.; Sparén, P.; Widmark, C. Interventions to improve cervical cancer screening uptake amongst young women: a systematic review. Acta Oncol. 2014, 53(4), 445-51. DOI: 10.3109/0284186X.2013.869618; PMID: 24660768.	Included
7	Arbyn. M.; Verdoodt, F.; Snijders, P.J.; Verhoef, V.M.; Suonio, E.; Dillner, L.; Minozzi, S.; Bellisario, C.; Banzi, R.; Zhao, F.H.; et al. Accuracy of human papillomavirus testing on self-collected versus clinician-collected samples: a meta-analysis. Lancet Oncol. 2014, 15(2), 172-83. DOI: 10.1016/S1470-2045(13)70570-9; PMID: 24433684.	Included
8	Morgan, K.; Azzani, M.; Khaing, S.L.; Wong, Y.L.; Su, T.T. Acceptability of Women Self-Sampling versus Clinician-Collected Samples for HPV DNA Testing: A Systematic Review. J. Low. Genit. Tract. Dis. 2019, 23(3), 193-199.	Included
9	Mezei, A.K.; Armstrong, H.L.; Pedersen, H.N.; Campos, N.G.; Mitchell, S.M.; Sekikubo, M.; Byamugisha, J.K.; Kim, J.J.; Bryan, S.; Ogilvie, G.S. Cost-effectiveness of cervical cancer screening methods in low- and middle-income countries: A systematic review. Int. J. Cancer 2017, 141(3), 437-446.	Included
10	Arbyn M., Castle P. E., Schiffman M. et al. (2022). Meta-analysis of agreement/concordance statistics in studies comparing self- vs	Included

	**clinician-collected samples for HPV testing in cervical cancer screening. Int. J. Cancer. 151(2): 308-312**	
**11**	**Kelly, H.; Mayaud, P.; Segondy, M.; Pant Pai, N.; Peeling, R.W. A systematic review and meta-analysis of studies evaluating the performance of point-of-care tests for human papillomavirus screening. Sex. Transm. Infect. 2017, 93(S4), S36-S45.**	**Included**
**12**	**Zhao, F.H.; Lewkowitz, A.K.; Chen, F.; Lin, M.J.; Hu, S.Y.; Zhang, X.; Pan, Q.J.; Ma, J.F.; Niyazi, M.; Li, C.Q.; et al. Pooled analysis of a self-sampling HPV DNA Test as a cervical cancer primary screening method. J. Natl. Cancer Inst. 2012, 104(3), 178-88.**	**Included**
**13**	**Racey, C.S.; Withrow, D.R.; Gesink, D. Self-collected HPV testing improves participation in cervical cancer screening: a systematic review and meta-analysis. Can. J. Public Health 2013, 104(2), e159-66.**	**Included**
**14**	**Yeh, P.T.; Kennedy, C.E.; De Vuyst, H.; Narasimhan, M. Self-sampling for human papillomavirus (HPV) testing: A systematic review and meta-Analysis. BMJ Glob. Health 2019, 4, e001351.**	**Included**
**15**	**Tesfahunei, H.A.; Ghebreyesus, M.S.; Assefa, D.G.; Zeleke, E.D.; Acam, J.; Joseph, M.; Getachew, E.; Kajogoo, V.D.; Bekele, D.; Manyazewal, T. Human papillomavirus self-sampling versus standard clinician-sampling for cervical cancer screening in sub-Saharan Africa: a systematic review and meta-analysis of randomized controlled trials. Infect. Agent. Cancer. 2021, 16(1): 43.**	**Included**
16	Tsiachristas A., Gittins M., Kitchener H. et al. (2018). Cost-effectiveness of strategies to increase cervical screening uptake at first invitation (STRATEGIC). J. Med. Screen. 25(2): 99-109	Excluded
17	Caleia A. I., Piresa C., de Fátima Pereira J. et al. (2020). Self-Sampling as a Plausible Alternative to Screen Cervical Cancer Precursor Lesions in a Population with Low Adherence to Screening: A Systematic Review. Acta Cytol. 64(4): 332-343	Excluded
18	Nodjikouambaye Z. A., Adawaye C., Bouassa R. S. et al. (2020). A systematic review of self- sampling for HPV testing in Africa. Int. J. Gynaecol. Obstet. 149(2): 123-129	Excluded
19	Marjolein Dieleman M., de Waard J., Bea G. et al. (2022). Preferences and Experiences Regarding the Use of the Self-Sampling Device in hrHPV Screening for Cervical Cancer. Patient. 15(2): 245-253	Excluded
20	Amir S. M., Idris I. B., Yusoff H. M. (2022). The Acceptance of Human Papillomavirus Self-Sampling Test among Muslim Women: A Systematic Review. Asian Pac. J. Cancer Prev. 23(3): 767-774	Excluded
21	Serrano B., Ibáñez R., Robles C. et al. (2022). Worldwide use of HPV self-sampling for cervical cancer screening. Prevent. Med. Volume 154, 106900	Excluded
22	Dzobo M., Dzinamaria T., Kgarosi K. et al. (2022). Human papillomavirus self-sampling for cervical cancer screening among women in sub-Saharan Africa: a scoping review protocol. BMJ Open. 12: e056140	Excluded

23	Nagendiram A., Bougher H., Banks J. et al. (2019). Australian women's self-perceived barriers to participation in cervical cancer screening: A systematic review. Health. Promot. J. Austr. 31(3): 343-353	Excluded
24	Nishimura H., Yeh P. T., Oguntade H. et al. (2021). HPV self-sampling for cervical cancer screening: a systematic review of values and preferences. BMJ Global Health. 6: e003743	Excluded
25	Rossi P. G., Baldacchini F., Ronco G. (2014). The possible effects on socio-economic inequalities of introducing HPV testing as primary test in cervical cancer screening programs. Front. Oncol. 4: 20	Excluded
26	Zheng R., Heller D. S. (2020). High-Risk Human Papillomavirus Identification in Precancerous Cervical Intraepithelial Lesions. J. Low. Genit. Tract. Dis. 24(2): 197-201	Excluded

AMSTAR2

The systematic reviews included in the analysis received the following ratings:

- high – Musa 2017, Arbyn 2014;
- low – Arbyn 2018, Camilloni 2013;
- critically low – Arbyn 2022, Tesfahunei 2021, Malone 2020, Morgan 2019, Yeh 2019, Kelly 2017, Mezei 2017, Nelson 2017, Verdoodt 2015, Albrow 2014, Racey 2013, Zhao 2012.

Publication	Item 2	Item 4	Item 7	Item 9	Item 11	Item 13	Item 15	Overall rating
Arbyn 2022 (MA)	Partial Yes	Partial Yes	No	No	Yes	No	No	**Critically Low**
Tesfahunei 2021 (MA)	Yes	Partial Yes	No	Yes	Yes	Yes	No	**Critically Low**
Malone 2020 (SR)	Partial Yes	Partial Yes	No	No	–	No	–	**Critically Low**
Morgan 2019 (SR)	No	Partial Yes	No	Partial Yes	–	No	–	**Critically Low**
Yeh 2019 (MA)	Yes	Yes	No	Yes	Yes	Yes	No	**Critically Low**
Arbyn 2018 (MA)	Partial Yes	Partial Yes	No	Yes	Yes	Yes	Yes	**Low**
Kelly 2017 (MA)	Partial Yes	Partial Yes	No	Partial Yes	Yes	Yes	No	**Critically Low**
Mezei 2017 (SR)	No	Yes	No	No	–	No	–	**Critically Low**
Musa 2017 (MA)	Yes	Yes	Yes	Yes	Yes	Yes	Yes	**High**
Nelson 2017 (MA)	Yes	Yes	Partial Yes	No	Yes	No	No	**Critically Low**

Publication	Item 2	Item 4	Item 7	Item 9	Item 11	Item 13	Item 15	Overall rating
Verdoodt 2015 (MA)	Partial Yes	Partial Yes	No	Yes	Yes	Yes	No	**Critically Low**
Albrow 2014 (SR)	No	Partial Yes	No	No	–	No	–	**Critically Low**
Arbyn 2014 (MA)	Partial Yes	Partial Yes	Yes	Yes	Yes	Yes	Yes	**High**
Camilloni 2013 (MA)	Partial Yes	Partial Yes	Yes	Yes	Yes	Yes	No	**Low**
Racey 2013 (MA)	No	Partial Yes	No	No	Yes	No	No	**Critically Low**
Zhao 2012 (MA)	No	No	No	No	No	No	No	**Critically Low**

MA – meta-analysis; SR – systematic review

Critical domains: item 2 – protocol registered before commencement of the review; item 4 – adequacy of the literaturę search; item 7 – justyfication for excluding individual studies; item 9 – risk of bias from individual studies being included in the review; item 11 – appropriateness of meta-analytical methods; item 13 – consideration of risk of bias when interpreting the results of the review; item 15 – assessment of presence and likely impact of publication bias.
